# Levodopa-induced dyskinesia is still a major clinical problem in Brazilian movement disorder clinics

**DOI:** 10.1055/s-0045-1806922

**Published:** 2025-05-20

**Authors:** Vitor Tumas, Manuelina Mariana Capellari Macruz Brito, Vanderci Borges, Henrique Ballalai Ferraz, Cyrus P. Zabetian, Ignacio F. Mata, Bruno Lopes Santos-Lobato

**Affiliations:** 1 Universidade de São Paulo, Faculdade de Medicina de Ribeirão Preto, Departamento de Neurociências e Ciências do Comportamento, Ribeirão Preto SP, Brazil.; 2 Universidade Federal de São Paulo, Escola Paulista de Medicina, Departamento de Neurologia, São Paulo SP, Brazil.; 3 Veterans Affairs Puget Sound Health Care System and University of Washington, Seattle, WA, United States.; 4 Genomic Medicine Institute, Lerner Research Institute, Cleveland Clinic, Cleveland OH, United States.; 5 Universidade Federal do Pará, Faculdade de Medicina, Laboratório de Neuropatologia Experimental, Belém PA, Brazil.

**Keywords:** Parkinson Disease, Dyskinesias, Levodopa, Brazil

## Abstract

**Background:**

Levodopa-induced dyskinesia (LID) remains a significant motor complication in Parkinson's disease (PD), although opinions differ on its clinical relevance.

**Objective:**

To explore the current prevalence and impact of LID, we analyzed two cohorts from the Latin American Research Consortium on the Genetics of Parkinson's Disease from movement disorder clinics in the city of São Paulo, Brazil, recruited 10 years apart.

**Methods:**

The cohorts included 187 individuals diagnosed with PD in phase 1 (2007–2014) and 224 in phase 2 (2021–2022). The presence and functional impact of LID were measured using part IV (items 4.1 and 4.2 respectively) of the Movement Disorder Society Unified Parkinson's Disease Rating Scale (MDS-UPDRS).

**Results:**

The analysis revealed that LID frequency increased from 34.7 in phase 1 to 54.9% in phase 2 (more recent), with functional impact rising from 25.1 to 38.8%.

**Conclusion:**

The findings suggest that LID remains a relevant clinical issue in clinics specialized in movement disorders in Brazil, with no reduction in prevalence throughout the last decade. Further studies from other regions and less specialized neurology centers may help understand this motor complication in Brazil and in other developing countries.

## INTRODUCTION

Levodopa-induced dyskinesia (LID) is one of the motor complications observed in people with Parkinson's disease (PD) during chronic treatment with levodopa. 1 It usually presents as predominantly choreiform and athetoid involuntary movements, frequently appearing a few minutes after taking levodopa, during the peak plasma concentration of the drug, and is called *peak-dose dyskinesia* . This condition was soon observed by the precursors in the clinical use of levodopa and, at that time, it was considered the most frequent and important side effect of chronic treatment with the drug. 2 In the early 1990s, LID was considered one of the most common and complex motor complications to manage in people with PD. 3

However, some authors 4 no longer consider LID a relevant clinical problem and, therefore, they believe it should not be a priority in the allocation of resources for research and the development of new treatments. The main reason for this opinion is the clinical impression that, over time, LID became a secondary problem. This could be partly attributed to changes in the prescription practice of levodopa, now using lower total daily doses and more organized therapeutic regimens, and the routine prescription of amantadine, which helps to control and prevent the worsening of LID. In more complicated cases, the indication of surgical treatment for the disease usually eliminates the problem. In a population-based cohort of PD patients in the United States treated preferentially with levodopa from the onset, 5 LID was present in only 30% of the subjects, and in only 3% of them the dyskinesias were severe. On the other hand, other authors 6 consider that LID is still a relevant clinical problem that needs to be better understood, and that it can have a significant impact on the lives of a considerable proportion of PD patients. They 6 argue that, although levodopa is used with caution, as the disease progresses, it will inevitably be necessary to take higher doses of the medication, which is the main risk factor for the onset of LID. Surgical treatment, considered the primary option to manage motor complications, is not indicated for most PD patients. In certain countries, such as Brazil, even people with PD with an indication for this procedure may not have access to it. The argument that most patients do not consider LID a significant problem is not shared by many clinicians, and this individual perception can vary between different cultures and countries. 7

Considering the actual debate about the current clinical importance of LID, we aimed to compare the prevalence and severity of LID in two different cohorts of Brazilian PD patients recruited 10 years apart from one another in the same specialized clinics and with the same inclusion and exclusion criteria. We also analyzed whether, throughout these 10 years, there would be a trend toward a reduction in the frequency of PD patients with problematic LID.

## METHODS

The Latin American Research Consortium on the Genetics of Parkinson's Disease (LARGE-PD) began in 2005 as a Latin American multicenter collaboration to increase knowledge about the genetics of PD in these countries. The inclusion criteria for the LARGE-PD studies were the same:

- consecutive PD patients cared for at the Movement Disorder Outpatient Clinic at Hospital das Clínicas da Faculdade de Medicina de Ribeirão Preto, Universidade de São Paulo (FMRP/USP) and the Extrapyramidal Outpatient Clinic at Hospital São Paulo (Universidade Federal de São Paulo, UNIFESP);
- patients diagnosed with PD according to the London Brain Bank criteria; and
- subjects older than 18 years of age.

The exclusion criteria were:

- not agreeing to sign the informed consent form to take part in the study; and
- being under 18 years of age.

The evaluation protocol included applying the Movement Disorder Society Unified Parkinson's Disease Rating Scale (MDS-UPDRS). We retrospectively analyzed protocols from people diagnosed with PD in two centers of the LARGE-PD study in Brazil. We divided the data into 2 recruitment periods: inclusion between 2007 and 2014 (phase 1; LARGE-PD1) and from 2021 to 2022 (phase 2; LARGE-PD2).

We considered the presence of LID when the PD patients scored ≥ 1 on part IV, item 4.1—time with dyskinesia, of the MDS-UPDRS; any significant functional impact of LID was considered when the PD patients scored > 1 on part IV, item 4.2—functional impact of dyskinesias, of the MDS-UPDRS.

We used the Student's t-test to compare variables with a normal distribution, and the non-parametric Mann-Whitney test to compare variables without a normal distribution. We used the Chi-squared test to check whether two categorical variables are related, and the Shapiro-Wilk test to check whether the samples were normally distributed.

To analyze the impact of LID in PD in 2 cohorts separated by 10 years, we performed a multivariate logistic regression with the frequency and functional impact of LID as dependent variables. The cohort (LARGE-PD1 and LARGE-PD2) was the independent variable, and sex, age at evaluation, disease duration, and use of dopaminergic agonists were covariables.

## RESULTS

Table 1 presents the demographic and clinical aspects of the patients in both cohorts. The patients in LARGE-PD2 were older, presented longer disease duration, more severe motor symptoms, and used levodopa more frequently and dopamine agonists less frequently. In LARGE-PD1, none of the patients underwent surgical treatment for PD, while in LARGE-PD2, 17 subjects had undergone deep brain stimulator (DBS) implantation.

**Table 1:** Demographic and clinical aspects of the PD patients in the two phases of the LARGE-PD study

Clinical variables	LARGE-PD1 (2007–2014)	LARGE-PD2 (2021–2022)	*p* -value
Sample size: n	187	224	
Mean age at evaluation (years)	58.7(±12.5)	62.8(±11.5)	**0.001**
Mean age at disease onset (years)	49.8(±13.3)	50.3(±13.8)	0.74
Sex (Male/Female): n	115/72	142/82	0.77
Use of levodopa: n (%)	154 (82.3)	221 (98.6)	**< 0.001**
Use of dopamine agonists: n (%)	110 (58.8)	56 (25)	**< 0.001**
Mean total score on part III of the MDS-UPDRS	32.7(±19.3)	40.7(±21.0)	**< 0.001**
Surgical treatment for PD: n (%)	0 (0)	17 (7.5)	NT
Presence of LID: n (%)*	65 (34.7)	123 (54.9)	**< 0.001**
LID with functional impact: n (%)**	46 (24.5)	87 (38.8)	**< 0.004**

Abbreviations: LID, levodopa-induced dyskinesia; MDS-UPDRS, Movement Disorder Society Unified Parkinson's Disease Rating Scale; NT, not tested; PD, Parkinson's disease.

Notes: Values in bold indicate *p* < 0.05; *score ≥ 1 on part IV, item 4.1–time with dyskinesia of the MDS-UPDRS; **score > 1 on part IV, item 4.2–functional impact of dyskinesias of the MDS-UPDRS.

**Figure 1:**
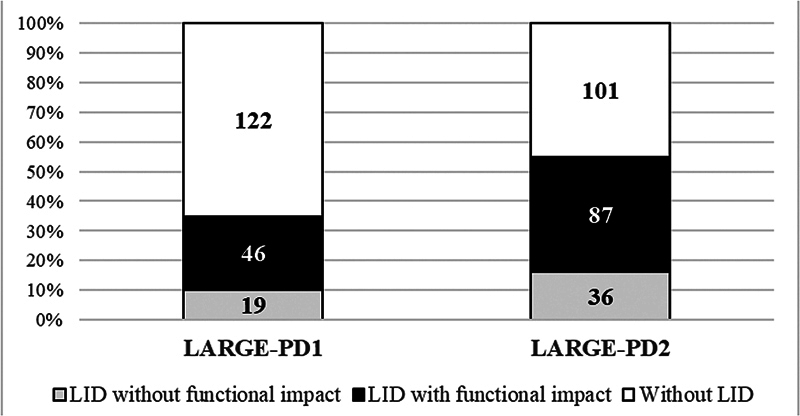
Visual representation of the frequency and functional impact of levodopa-induced dyskinesia (LID) on the two cohorts (LARGE-PD1 and LARGE-PD 2). The absolute number of participants in each cohort is represented inside bars.

In LARGE-PD1, 65/187 (34.7%) subjects presented LID, with functional impact on 25.1% of them. In LARGE-PD2, 123/224 (54.9%) subjects presented LID, with functional impact on 38.8%. Figure 1 shows the frequency and functional impact of LID on the two cohorts. Table 2 shows the distribution of MDS-UPDRS scores associated with LID (items 4.1 and 4.2) in both cohorts.

**Table 2:** Distribution of scores on part IV, items 4.1 and 4.2, of the MDS-UPDRS of the PD patients in the 2 phases of the LARGE-PD study

**LARGE-PD1 (2007–2014)**							
		**Item 4.2 (functional impact of dyskinesias)**					
		**0**	**1**	**2**	**3**	**4**	
**Item 4.1 (time with dyskinesias)**	**1**	16	15	3	1	1	**36 (55.3)**
	**2**	1	6	1	5	1	**14 (21.5)**
	**3**	0	3	2	1	2	**8 (12.3)**
	**4**	2	2	2	1	0	**7 (10.7)**
		**19 (29.2)**	**26 (40)**	**8 (12.3)**	**8 (12.3)**	**4 (6.1)**	**65**
**LARGE-PD2 (2021–2022)**							
		**Item 4.2 (functional impact of dyskinesias)**					
		**0**	**1**	**2**	**3**	**4**	
**Item 4.1 (time with dyskinesias)**	**1**	32	29	11	7	1	**80 (65)**
	**2**	4	7	7	4	0	**22 (17.8)**
	**3**	0	2	1	7	1	**11 (8.9)**
	**4**	0	3	4	3	0	**10 (8.1)**
		**36 (29.2)**	**41 (33.3)**	**23 (18.6)**	**21 (17)**	**2 (1.6)**	**123**

Abbreviation: MDS-UPDRS, Movement Disorder Society Unified Parkinson's Disease Rating Scale.

Note: The values in parentheses indicate the proportion of people with each score. The number in bold indicates the number (%).

Regarding the risk of developing LID, the multivariate logistic regression analysis showed that being part of the more recent cohort (LARGE-PD2; adjusted odds ratio [OR] = 2.08; 95% confidence interval [95%CI] = 1.30–3.30; *p* = 0.002) and presenting a longer disease duration (adjusted OR = 1.10; 95%CI = 1.07–1.14; *p* < 0.001) increased the chances of developing LID, regardless of gender, age at assessment, and use of dopamine agonists. There was no association involving functional impact and other variables ( Table 3 ).

**Table 3:** Results of multivariate logistic regression analysis with the frequency and functional impact of LID as dependent variables, were the cohort (LARGE-PD1 and LARGE-PD2) was the independent variable, and sex, age at evaluation, disease duration, and use of dopaminergic agonists were covariables

Clinical variables	n	B	aOR	95%CI	*p* -value
**Frequency of levodopa-induced dyskinesias**					
**LARGE-PD2 cohort**	395	0.733	2.08	1.30-3.30	0.002
**Male sex**	395	−0.021	0.92	0.63-1.52	0.979
**Age at evaluation**	395	−0.012	0.98	0.97-1.00	0.200
**Disease duration**	395	0.103	1.10	1.07-1.14	< 0.001
**Use of dopaminergic agonists**	395	0.268	1.30	0.81-2.08	0.262
**Functional impact of levodopa-induced dyskinesias**					
**LARGE-PD2 cohort**	181	0.311	1.36	0.68-2.73	0.382
**Male sex**	181	−0.184	0.83	0.44-1.56	0.568
**Age at evaluation**	181	0.015	1.01	0.98-1.04	0.299
**Disease duration**	181	0.027	1.02	0.98-1.07	0.224
**Use of dopaminergic agonists**	181	0.219	1.24	0.63-2.43	0.522

Abbreviations: 95%CI, 95% confidence interval; aOR, adjusted odds ratio; B, regression coefficient.

Note: Values in bold numbers indicate *p* < 0.05.

## DISCUSSION

According to the data of the present study, LID remains a common and significant clinical problem in movement disorder clinics in São Paulo, Brazil's most populated and wealthiest state. From the first to the second phases, the frequency of LID increased, and the functional impact of dyskinetic movements remained stable.

Regarding the increased frequency of LID between the two periods, our small sample may underpower our conclusions. The higher proportion of patients with longer disease duration and greater severity of motor symptoms in LARGE-PD2, both known clinical risk factors associated with LID onset, could explain the higher frequency of LID in this group. Our analysis showed that LID remains a common clinical problem in these specialized clinics, and it even indicates that the condition has become more common over the last decade in these sites.

Regarding the impact of LID on the functionality of PD patients, LARGE-PD2 showed that 8% of the subjects had LID for > 75% of the waking day (score of 4 on part IV, item 4.1, of the MDS-UPDRS), and only 1.6% had problematic LID (score of 4 on part IV, item 4.2, of the MDS-UPDRS IV). However, if we consider scores of 3 and 4 on items 4.1 and 4.2 for this group, 17% had LID for > 50% of the waking day, and 18.6% had moderate or severe LID. Thus, these results suggest that 1 in every 5 patients with PD and LID from specialized movement disorder clinics in Brazil may still present troublesome dyskinesias. These data are similar to those observed in another Brazilian cohort from a specialized clinic in Southern Brazil. 8 In another recent analysis, a large cohort of PD patients in Spain also showed that the frequency of LID increases significantly with longitudinal follow-up, and that it causes significant impairment in the patients' quality of life. 9

The reasons why the frequency of LID increased over the years in these specialized Brazilian centers are unclear. It is possible that these observations are simply the result of a referral bias. Although access to surgical treatment for PD is restricted in poorer countries like Brazil, we observed an apparent increase in the number of PD patients operated in our sample. Our findings suggest that the demand for surgery is greater than the availability, and that expanding the capacity to tend to the needs of more complicated patients is necessary.

The comparative analysis of the two recruitment periods revealed a change in the treatment profile of PD patients: those assessed more recently were treated more frequently with levodopa and less frequently with dopamine agonists. This difference could be partly explained by the greater severity of motor symptoms and the older age of the PD patients included in LARGE-PD2. This recent change in the treatment profile agrees with the suggested management strategy disseminated and reinforced more recently for advanced PD.

A few years ago, the prescription of dopamine agonists was advocated as a strategy to delay or spare the use of levodopa in the treatment of PD. Nowadays, many authors argue that dopamine agonists have significant side effects, causing drowsiness and especially impulse control disorders; therefore, they should be avoided, if possible. This strategy requires higher doses of levodopa for motor symptom management, which may increase the cases of onset of LID. This may have been one of the reasons for the increased frequency of LID in the patients in LARGE-PD2.

However, the limitations of the data in the present study do not enable us to reach a clear conclusion on this. It is not possible to clearly establish how treatment strategies have changed in these centers. We have no information about the doses of levodopa and dopamine agonists used, nor about the use of amantadine by these patients. The Brazilian government has a program to provide these drugs free of charge, so, theoretically, there would be no restrictions to the adoption by clinicians of any priority treatment strategy or to the indication to use amantadine, which can reduce and even prevent LID.

As limitations, it must be considered that the clinical assessment of LID using only part IV of the MDS-UPDRS is limited. It does not adequately consider the subjective perception of discomfort, shame, and embarrassment that involuntary movements can cause in each person with PD. A more focused and detailed assessment, such as the Unified Dyskinesia Rating Scale, 10 may better measure how much LID can impact patients' daily lives. Moreover, the lack of data from other Brazilian regions and less specialized neurology centers impairs the generalizability of these findings.

The study has strong points which must be highlighted. The PD patients assessed in the two phases of the LARGE-PD study were subjected to the same inclusion and exclusion criteria, and all were thoroughly evaluated by examiners trained to apply the MDS-UPDRS. The PD patients were also evaluated at the same recruitment centers supervised by the same principal investigators.

The LARGE-PD study is a large Latin American multicenter project that will collect various clinical and environmental exposure data and genotype many people with PD in Latin America. This information will enable the conduction of other important analyses on LID in this population, such as that of the associations with therapeutic regimens, individual factors, environmental exposure, and genetic factors. Thus, we may have better insight into the mechanisms and risk factors involved in the onset of LID in Latin American PD patients.

In conclusion, LID is still very common and clinically relevant in specialized movement disorder clinics in Brazil, and there is no evidence that its prevalence has reduced over the years. Further studies from other regions without movement disorder clinics may help understand this motor complication in Brazil and in other developing countries.
