# Erratum

**DOI:** 10.1055/s-0045-1809947

**Published:** 2025-08-25

In the article “Levodopa-induced dyskinesia is still a major clinical problem in Brazilian movement disorder clinics”, with DOI code number https://doi.org/10.1055/s-0045-1806922 , published in the Arq Neuropsiquiatr. 2025 Apr;83(4):1-5.

**The authors' affiliation:**

^3^ University of Washington, Department of Neurology, Seattle WA, United States.

**Should be:**

^3^ Veterans Affairs Puget Sound Health Care System and University of Washington, Seattle, WA, United States.

